# Risk factors for bone mineral density at the calcaneus in 40–59 year-old male workers: A cross-sectional study in Korea

**DOI:** 10.1186/1471-2458-8-253

**Published:** 2008-07-23

**Authors:** Hyun-Ju Seo, Soo-Geun Kim, Chong-Soon Kim

**Affiliations:** 1 Department of Public Health, Graduate School of Korea University, 126-1, 5-ka, Anam-dong, Sungbuk-Gu, Seoul, 136-705, Korea; 2 Department of Occupational Medicine, Kangbuk Samsung Hospital, School of Medicine, Sungkyunkwan University, 108, Pyung-dong, Jongno-Gu, Seoul, 110-746, Korea; 3 Korea Institute of Radiological & Medical Sciences, 215-4, Gongneung-Dong, Nowon-Gu, Seoul 139-706, Korea

## Abstract

**Background:**

Few epidemiologic studies have attempted to investigate the prevalence and risk factors for osteopenia and osteoporosis in middle-aged Asian men. We performed this study to determine the prevalence and risk factors of osteopenia and osteoporosis in this population.

**Methods:**

This cross-sectional study was conducted from March to July, 2004. The subjects were 2,073 males aged from 40 to 59 years in the KHNP (Korea Hydro & Nuclear Power) workplace-based cohort. Bone mineral density (BMD) was measured by peripheral, dual-energy, X-ray absorptiometry (DXA) at the calcaneus. Anthropometric and lifestyle factors were investigated using a standard, self-reported questionnaire.

**Results:**

BMD was 0.60 ± 0.09 g/cm^2 ^(mean ± standard deviation) and was negatively correlated with age (r = -0.18, *P *< 0.001), but positively correlated with waist-to-hip ratio (WHR; r = 0.15, *P *< 0.001), body fat (r = 0.10, *P *< 0.001), BMI (r = 0.35, *P *< 0.001), height (r = 0.26, *P *< 0.001), and weight (r = 0.43, *P *< 0.001).

In multiple linear regression analysis, the independent determinants associated with BMD were increasing age (coefficient = -0.002, *P *< 0.001), physical activity (≤ 2/week vs. ≥ 3/week; coefficient = 0.017, *P *< 0.001), WHR (coefficient = -0.796, *P *< 0.001), body mass index (BMI; coefficient = 0.023, *P *< 0.001) and smoking status (never vs. ever; coefficient = -0.018, *P *< 0.001).

**Conclusion:**

We suggest that BMD of the calcaneus is correlated negatively with exposure to smoke and increased WHR, but positively with regular exercise and increased BMI.

## Background

WHO recognizes osteoporosis as an important, global health problem that will increase in significance as the world population both increases and ages [1,2]. Since most of the lifestyle aspects that affect osteoporosis are modifiable, lifestyle modification is important in the prevention of osteoporosis.

In recent studies, the prevalence of osteoporosis in men older than 49 years old was about 7% [3], and fatalities caused by femoral neck fracture were more common in men than in women [4]. In addition, osteoporosis in elderly men has become an important disease because one study found that 25% of men were in danger of fracture due to osteoporosis [5]. Moreover, a quick recovery from osteoporosis is not possible and osteoporosis increases the fracture risk [6], therefore increasing the importance of prompt treatment through prevention and early diagnosis. Therefore, the quality of life in Korea, which is an aging society, can be improved by focusing on the prevention of osteoporosis in the general population.

Previous studies of osteoporosis examined the risk factors of osteoporosis [7-9], exercise and bone density [10,11] and calcium and bone density [12,13]. However, the study subjects were American or European women. Studies of lifestyles related to osteoporosis in women have been performed, especially climacteric women [14] and university and college students [15], but the epidemiological conditions and risk factors of osteoporosis in Korean men remain to be elucidated due to the absence of any studies of osteoporosis in Korean men. Moreover, most subjects of domestic and foreign studies were older than 47 years old [16-23]. Therefore, the aim of this study was to assess the prevalence of osteoporosis, which is a major health problem, in 40–59 year-old male workers, and to obtain epidemiological data for the association between life style factors and bone density.

## Methods

### Subjects and methods

The subjects comprised 2,073 male workers with age ranging from 40 to 59 at five hydroelectric and nuclear power plants operated by Korea Hydro & Nuclear Power (KHNP) company at Kori, Yonggwang, Ulchin, Wolsong, and Seoul in Korea.

The KHNP cohort inspected the workers working for the nuclear power plants. The aim of this survey was to examine the impact of exposure to low-dose radiation on the employees' health status. In 2004, 6,980 workers, with an average age of 39.8 ± 8.3 years, underwent an annual health check-up.

The 40–59 year-old men comprised 47.0% of this population. We conducted epidemiology research at five sites from March to July, 2004, and enrolled 2,073 subjects who agreed to our survey among the 3,275 total. The study was designed to analyze the database; health examinations' data of KHNP workers on the condition of secrecy under worker's information. Therefore the study was exempted from a review of the Institutional Review Board at a point of its planning time since it was the observational study for academic purpose using an existing dataset that did not involve personal information under the exemption criteria. The data deleted the name and citizen registration number of each worker was provided. Researchers only accessed to analyze the database.

All participants agreed with written informed consent described the purpose of the establishment of a program for health promotion about diseases related with lifestyles.

We measured the bone mineral density (BMD), body composition (body fat percentage, waist-to-hip ratio (WHR)), height, and weight. To collect data on lifestyles, we investigated educational levels, smoking status, drinking status, and frequency of physical activity through standard, self-reported questionnaires.

For examination of body composition, we used Inbody 3.0 of Biospace Co. to measure body fat percentage and WHR, the latter via bioimpedance measurement [24].

### Bone mineral density (*BMD*) measurement

BMD was assessed by measurements taken at the calcaneus by dual-energy X-ray absorptiometry (DXA) using an EXA-3000 (Osteosys, Seoul, Korea), according to the protocol (precision error; <1.0% CV in vivo). Quality control procedures were carried out in accordance with the manufacturer's guidance.

We measured the bone density at the calcaneus, which has been validated as a measurement site and is considered to be highly predictive of fracture risk [25]. In addition, this peripheral densitometry device has the advantages of low cost and portability for field epidemiologic study of osteoporosis.

BMD measurements provided absolute values for the calcaneus site and were then compared to those of healthy young Korean adults (T score). The reference population was 81 female and 81 male subjects, as provided by the manufacturer of the bone densitometry.

### Statistical analysis

The results are presented as means (± SD) and categorical variables are expressed as frequencies. We used Pearson's correlation coefficient to examine the effect of continuous variables on BMD, and performed multiple linear regression analysis to determine the independent effect of variables related with BMD. To examine the multi-collinearity of the regression model, we checked the variance inflation factor. A variance inflation factor greater than 10 indicates that the model is problematic [26]. Associations were considered statistically significant at the p < 0.05 level. The SPSS 12.0 (for window) statistical software package was used for statistical analysis.

## Results

The general characteristics of study subjects are shown in Table 1. The mean age of the subjects was 47.1 years old, and the mean BMD was 0.60 ± 0.09 g/cm^2^.

**Table 1:** General characteristics of the study subjects (n = 2,073)

Variables	Mean ± SD
Age(year)	47.1 ± 4.8
Height(cm)	169.5 ± 5.4
Weight(kg)	69.6 ± 8.4
BMI(kg/m^2^)	24.2 ± 2.5
Body fat(%)	21.3 ± 4.2
Waist hip ratio	0.9 ± 0.1
Bone mineral density (g/cm^2^)	0.60 ± 0.09
Education level^✝^ (year)	
≤ 12	884 (42.6)
>12	1,189 (57.4)
Smoking status^✝^	
never-smoker	473 (22.8)
ever-smoker	1,600 (77.2)
Drinking status^✝^	
never-drinker	191 (9.2)
ever-drinker	1882 (90.8)
Physical activity^✝^ (time/week)	
≤ 2	1,038 (50.1)
≥ 3	1,035 (49.9)

Bone mineral density grouping(n(%))*	
normal group	1,539 (74.2)
osteopenia	472 (22.8)
osteoporosis	62 (3.0)

* Bone mineral density grouping: according to diagnostic criteria of WHO, normal group; T-score ≥ -1.0, osteopenia; -2.5 ≤ T-score < -1.0, osteoporosis; T-score < -2.5

^✝^ n(%)

Correlation analysis was conducted to investigate the continuous variables related with BMD. BMD was correlated negatively with age (r = -0.18, *P *< 0.001), but positively with WHR (r = 0.15, *P *< 0.001), body fat (r = 0.10, *P *< 0.001), height (r = 0.26, *P *< 0.001), and weight (r = 0.43, *P *< 0.001) (Table 2).

**Table 2:** Correlation between various parameters and bone mineral density

	*r*	*P*
Age (year)	-0.18	<0.001
WHR	0.15	<0.001
Body fat (%)	0.10	<0.001
BMI (kg/m^2^)	0.35	<0.001
Height (cm)	0.26	<0.001
Weight (Kg)	0.43	<0.001

Pearson correlation coefficient (*r*)

Multiple linear regression analysis was performed to identify the related factors that affect BMD. Age, education level (<12 years vs. ≥ 12 years), physical activity (≤ 2/week vs. ≥ 3/week), WHR, BMI, drinking status (never vs. ever), and smoking status (never vs. ever) were selected from those subjects scoring less than 10.0 in the variance inflation factors, i.e., body fat, height and weight were excluded.

The independent parameters associated with BMD were age (coefficient = -0.002, *P *< 0.001), physical activity (≤ 2/week vs. ≥ 3/week; coefficient = 0.017, *P *< 0.001), WHR (coefficient = -0.796, *P *< 0.001), BMI (coefficient = 0.023, *P *< 0.001) and smoking status (never vs. ever; coefficient = -0.018, *P *< 0.001). The variance inflation factors in this regression model were less than 4.01 and the adjusted R^2 ^value was 20.7% (Table 3).

**Table 3:** Multiple linear regression analysis on variables associated with bone mineral density

	Coefficient	Standard error	t	P*
Age (year)	-0.002	0.000	-5.522	<0.001
Education level (<12 years vs. ≥ 12 years)	0.001	0.004	0.301	0.763
Physical activity (≤ 2/week vs. ≥ 3/week)	0.017	0.003	4.895	<0.001
WHR	-0.796	0.093	-8.569	<0.001
BMI (kg/m^2^)	0.023	0.001	17.033	<0.001
Drinking status (never vs. ever)	-0.002	0.006	-0.259	0.795
Smoking status (never vs. ever)	-0.018	0.004	-4.317	<0.001

*P < 0.05 by multiple linear regression analysis, R^2 ^= 0.21

## Discussion

Osteoporosis is a cause of significant morbidity and mortality in both postmenopausal women and men [27]. At present, there are no sufficient data for epidemiological research on the bone density of healthy, middle-aged, male workers in Korea.

In a study with 152 healthy, middle-aged men [28], the prevalence of osteoporosis and osteopenia in the lumbar vertebra was 3.9% and 28.3%, respectively. In a study that investigated the bone density of the femoral neck of American men older than 49 years old, the prevalence of osteoporosis was 3~6% and that of osteopenia 28~47% [29]. In a study of Canadian men older than 49 years old, the prevalence of osteoporosis in the lumbar vertebra and femoral neck was 2.9% and 4.8%, respectively, giving a total of 6.6% [30]. In the present study, the prevalence of osteoporosis in the calcaneus was 3.0% and that of osteopenia was 22.8%, according to the diagnostic criteria of WHO.

Several studies have reported physical activity to be a relevant factor of osteoporosis [10,31,32]. Rikli & McManics [33] reported that weight load exercise was an effective training form. Hsu et al [34] reported that vigorous physical activity decreased osteopenia by 0.87-fold and osteoporosis by 0.74-fold. Consistent with these results of previous studies, physical activity (people who exercised three times or more a week) in the present study was positively associated with BMD.

Tobacco exposure has been implicated as a risk factor for decreased bone density, which might result in osteoporosis. Similar to previous studies, we observed negative associations between the smoking exposure and BMD. Byron and Jay [35] suggested that serum cotinine, as a marker for tobacco exposure, is a significant risk factor for decreased bone mineral content. In addition, the bone density of smokers may be low because smokers lack calcium uptake or tend to exercise less than never smokers [36]. In a study of 410 people aged from 61 to 73, the density of the lumbar vertebra of smokers was lower than that of never smokers [37]. In middle-aged men, there was a negative correlation between history of smoking and BMD, and this correlation was especially strong in current smokers [17,20,38].

Consistent with previous studies, there was a negative association between BMD and age. The most powerful predictor of osteoporosis was increased age [39,40]. Eastell et al [41] reported that age-induced decrease of bone density could be the result of decrease of kidney function, deficiency of vitamin D, increase of parathyroid hormone, decrease of testosterone or decrease of both calcium uptake and absorption. Moreover, two studies reported that the odds ratio for fracture in men with osteoporosis was 2–2.7 compared to men with normal bone density, indicating that decreased bone density in men was also associated with an increased risk for fracture [42,43].

BMI and WHR were used as parameters of general obesity and fat distribution, respectively. BMI was positively related to BMD, whereas WHR was inversely associated with BMD. Our results are consistent with those from other studies presenting a positive association of BMD with BMI [16,17,19,41,44-46] and WHR [32,47,48].

We suggest that BMD of the calcaneus is associated negatively with smoke exposure and increased WHR, but positively with regular exercise and increased BMI.

The study limitation was that the workplace-based participants may not truly represent the general Korean population due to the selection bias known as the healthy worker effect.

## Conclusion

This research provided epidemiological data on the BMD of Korean middle-aged men. We suggest that among 40 to 59 year-old male workers, BMD is negatively related to smoke exposure and increased WHR, but positively with regularly physical activity and increased BMI.

## Competing interests

The authors declare that they have no competing interests.

## Authors' contributions

H–JS performed the data analysis, and drafted and revised the manuscript. S–GK was responsible for the study design and helped to draft the manuscript. C–SK gathered the data and helped to draft the manuscript. All authors have read and approved the final manuscript.

## Pre-publication history

The pre-publication history for this paper can be accessed here:


